# Arsenic Species in Chicken Breast: Temporal Variations of Metabolites, Elimination Kinetics, and Residual Concentrations

**DOI:** 10.1289/ehp.1510530

**Published:** 2016-03-18

**Authors:** Qingqing Liu, Hanyong Peng, Xiufen Lu, Martin J. Zuidhof, Xing-Fang Li, X. Chris Le

**Affiliations:** 1 Department of Laboratory Medicine and Pathology, Faculty of Medicine and Dentistry, and; 2 Department of Agricultural, Food and Nutritional Science, Faculty of Agricultural, Life and Environmental Sciences, University of Alberta, Edmonton, Alberta, Canada

## Abstract

**Background:**

Chicken meat has the highest per capita consumption among all meat types in North America. The practice of feeding 3-nitro-4-hydroxyphenylarsonic acid (Roxarsone, Rox) to chickens lasted for more than 60 years. However, the fate of Rox and arsenic metabolites remaining in chicken are poorly understood.

**Objectives:**

We aimed to determine the elimination of Rox and metabolites from chickens and quantify the remaining arsenic species in chicken meat, providing necessary information for meaningful exposure assessment.

**Methods:**

We have conducted a 35-day feeding experiment involving 1,600 chickens, of which half were control and the other half were fed a Rox-supplemented diet for the first 28 days and then a Rox-free diet for the final 7 days. We quantified the concentrations of individual arsenic species in the breast meat of 229 chickens.

**Results:**

Rox, arsenobetaine, arsenite, monomethylarsonic acid, dimethylarsinic acid, and a new arsenic metabolite, were detected in breast meat from chickens fed Rox. The concentrations of arsenic species, except arsenobetaine, were significantly higher in the Rox-fed than in the control chickens. The half-lives of elimination of these arsenic species were 0.4–1 day. Seven days after termination of Rox feeding, the concentrations of arsenite (3.1 μg/kg), Rox (0.4 μg/kg), and a new arsenic metabolite (0.8 μg/kg) were significantly higher in the Rox-fed chickens than in the control.

**Conclusion:**

Feeding of Rox to chickens increased the concentrations of five arsenic species in breast meat. Although most arsenic species were excreted rapidly when the feeding of Rox stopped, arsenic species remaining in the Rox-fed chickens were higher than the background levels.

**Citation:**

Liu Q, Peng H, Lu X, Zuidhof MJ, Li XF, Le XC. 2016. Arsenic species in chicken breast: temporal variations of metabolites, elimination kinetics, and residual concentrations. Environ Health Perspect 124:1174–1181; http://dx.doi.org/10.1289/ehp.1510530

## Introduction

Since 1944 when the United States Food and Drug Administration (FDA) first approved the use of 3-nitro-4-hydroxyphenylarsonic acid (Roxarsone, Rox) as an animal feed additive, this organoarsenic compound has been extensively used in the poultry industry for more than 60 years to alleviate coccidiosis, promote growth and weight gain, and improve pigmentation of chickens (Chapman and Johnson 2002; Kowalski and Reid 1975; FDA 2015b). However, there have been considerable concerns over the use of Rox because of potential human exposure to arsenic species through the consumption of chicken (Conklin et al. 2012; FDA 2011; Lasky et al. 2004; Lasky 2013; Nachman et al. 2013). From 1999, the European Union ceased the use of arsenicals as feed additives (European Commission 1999). In 2011, an FDA study reported that the increased concentrations of inorganic arsenicals in chicken livers were attributed to feeding boiler chickens with Rox (FDA 2011). In response to the FDA study, the manufacturer of Rox in the United States has voluntarily suspended its supplies (FDA 2015b). In 2013, the FDA withdrew the approval of Rox (FDA 2013). However, Rox continues to be legally used in many other countries (Huang et al. 2014; Yao et al. 2013).

Although several studies have reported on the concentration of arsenic in Rox-fed chickens or in chicken meat purchased from food markets (Batista et al. 2012; Doyle and Spaulding 1978; Jelinek and Corneliussen 1977; Lasky et al. 2004), the information on the specific arsenic species is limited (Mao et al. 2011; Pizarro et al. 2003; Polatajko and Szpunar 2004; Sánchez-Rodas et al. 2006; Sanz et al. 2005). Determining the concentrations of individual arsenic species is important because the toxicity of arsenic is highly dependent on its chemical species. The median lethal concentrations of arsenic species vary by several orders of magnitude from the most toxic to the least toxic arsenic species (Charoensuk et al. 2009; Naranmandura et al. 2011; Shen et al. 2013; Styblo et al. 2000). Though Rox itself is of low toxicity to the test animals (Sullivan and Al-Timimi 1972), its toxicity to humans is not well understood. Furthermore, it is not clear how much other arsenic metabolites may be produced in Rox-fed chicken. It is crucial to determine the magnitude of increases in the concentrations of the more toxic arsenic species [e.g., arsenite (As^III^)] (Naujokas et al. 2013; IARC 2012).

Chicken is the number one meat consumed in North America on a per capita basis, with a supply of 17.7 billion kg per year (AAFC 2013; USDA 2014). It is paramount to assess the concentrations of individual arsenic species in this highly consumed food. The information will enable the assessment of human exposure to arsenic species and determine the relative contributions of arsenic species from the various sources.

Information on the metabolism of Rox in chicken is very limited (Conklin et al. 2012; FDA 2011; Overby and Straube 1965; Peng et al. 2014). Accurately identifying and quantifying arsenic species in chicken meat is challenging due to low concentrations of arsenic species. Therefore, previous work has often focused on chicken livers and feces that contain higher concentrations of arsenic species (Conklin et al. 2012; Falnoga et al. 2000; FDA 2011; Peng et al. 2014; Rosal et al. 2005; Salisbury et al. 1991). Recent work of Nachman et al. (2013) determined arsenic species in chicken samples collected in a U.S.-based market basket survey. This study found the concentrations of inorganic arsenicals were higher in conventional chickens [geometric mean (GM) = 1.8 μg/kg; 95% confidence interval (CI): 1.4, 2.3] than in antibiotic-free (GM = 0.7 μg/kg; 95% CI: 0.5, 1.0) or organic (GM = 0.6 μg/kg; 95% CI: 0.5, 0.8) chickens. The study also found a correlation between the higher concentrations of inorganic arsenicals (GM = 2.3 μg/kg; 95% CI: 1.7, 3.1) in the presence of Rox in the chicken samples compared to the concentrations of inorganic arsenicals (GM = 0.8 μg/kg; 95% CI: 0.7, 1.0) in Rox-negative samples. This correlation suggests that feeding of Rox may increase concentrations of As^III^ in chicken meat. This finding, together with the 2011 U.S. FDA study (FDA 2011), suggests that Rox may be partially biotransformed to inorganic arsenicals in the chicken body. However, it is still unknown whether feeding of Rox increases concentrations of other arsenic species in chicken meat. Moreover, how these arsenic species changed with the age of the chickens that were fed Rox remains a question.

To fill the knowledge gap, our research group has initiated a controlled feeding study that involved 1,600 chickens of two common commercial strains. In the first 4 weeks, half of the chickens (800) were fed a diet supplemented with Rox and the other 800 chickens were fed a control diet. This design allowed us to study the uptake and metabolism of Rox. In the final week, all chickens were fed a Rox-free diet, which allowed us to study the elimination kinetics over a 7-day period. We determined whether the feeding of Rox increased arsenic metabolites [e.g., arsenite and dimethylarsinic acid (DMA^V^)] in chicken breasts and the degree to which arsenic metabolites were eliminated from chicken breast meat after the feeding of Rox stopped.

## Methods

### Chicken Breast Meat Samples

Samples of chicken breast meat were collected from a 35-day poultry feeding study that was conducted at the Poultry Research Centre, University of Alberta. A total of 1,600 chickens (mixed sex), of two commercial broiler strains (Ross 308 and Cobb 500) were used. These 1,600 chickens were equally divided into the Rox-fed group and the control group. The controls (*n* = 800) were randomly divided, housed in eight pens (100 chickens per pen; 14.5 birds/m^2^), and fed a basal diet that was not supplemented with Roxarsone throughout the entire 5-week feeding period. The basal (control) diet had trace concentrations of arsenobetaine [(AsB) 0.03–0.1 μg/g], arsenate (As^V^; 0.04–0.1 μg/g), and DMA^V^ (0.03–0.04 μg/g), and no detectable As^III^ or monomethylarsonic acid (MMA^V^). The presence of AsB was due to the inclusion of menhaden fish meal as a protein source in the feed. The Rox-fed treatment group consisted of another 800 chickens, randomly allocated to another eight pens (100 chickens per pen; 14.5 birds/m^2^), and fed a Roxarsone-supplemented diet during the first 28 days (4 weeks), and the basal diet during the last week (day 29–35). The Roxarsone-supplemented diet was prepared from the basal diet with the addition of Roxarsone (18 ± 1 μg/g measured as arsenic), a standard supplementation dose commonly used in poultry practice (FDA 2015a). The last week of feeding without Roxarsone supplementation exceeded FDA regulations of withdrawal of Roxarsone for 5 days prior to processing in order to allow elimination of arsenic from the chicken bodies (FDA 2015a). Tap water from the same source in Edmonton (EPCOR, Edmonton, Alberta, Canada) (< 1 μg/L arsenic) was available to all the chickens throughout the entire 35-day period. Birds were provided a comfortable environment, with temperature set points decreasing linearly from 34°C on day 0 to 20°C by day 28, where temperature was maintained for the duration of the study. Twenty-three hours of light per day was provided for the first 3 days, which was reduced to 20 hr per day for the duration of the study. Males and females were housed together at random proportions, as the sex of chicks was not determined at hatching. On days 0, 1, 2, 3, 4, 7, 14, 21, 28, 29, 30, 31, 32, 33, 34, and 35, sixteen chickens were randomly selected (1 from each control and each Rox-fed pen, of random sex), euthanized by cervical dislocation, weighed, and the breast meat was collected. The sex of birds was determined visually upon dissection. Raw samples were stored at –80°C. Unfortunately, a few labels came off the sampling bag after freezing. To maintain integrity of the samples, we discarded any samples with questionable labeling. As a consequence, we analyzed 11–16 samples from each of the 16 sampling days, for a total of 229 samples.

All procedures involving animals were reviewed and approved by the University of Alberta Animal Care and Use Committee: Livestock (protocol #094). The feeding design and the age of chickens at breast sample collection are summarized in Table 1.

**Table 1:** Summary of the feeding experiment design and time of sample collection.

Feeding design						Age (days) at breast sample collection
Broiler strain	Group	Starter period (Day 0–14)	Grower period (Day 15–28)	Withdrawal period (Day 29–35)	*n* (chickens/pens)	
Ross 308	Rox-fed	Rox-supplemented diet	Rox-supplemented diet	Rox-free diet	400/4	0, 1, 2, 3, 4, 7, 14, 21, 28, 29, 30, 31, 32, 33, 34, 35
	Control	Rox-free diet	Rox-free diet	Rox-free diet	400/4	
Cobb 500	Rox-fed	Rox-supplemented diet	Rox-supplemented diet	Rox-free diet	400/4	
	Control	Rox-free diet	Rox-free diet	Rox-free diet	400/4	

### Determination of Arsenic Species

We analyzed all 229 chicken breast samples (114 from the control chickens and 115 from the Rox-fed chickens) for arsenic speciation using a previously developed method (Liu et al. 2015). Briefly, arsenic species in 0.5 g of freeze-dried samples were extracted using an enzyme-assisted extraction method, and each extract was analyzed in duplicate for arsenic speciation using high-performance liquid chromatography–inductively coupled plasma mass spectrometry (HPLC-ICPMS). Identities of arsenic species were confirmed using HPLC separation with simultaneous detection by ICPMS and electrospray ionization mass spectrometry. Detailed analytical procedures are included in Supplemental Material (“Analytical Procedures”) and the method evaluation has been described previously (Liu et al. 2015; Peng et al. 2014).

The limit of detection (LOD), obtained according to the method of the U.S. Environmental Protection Agency (EPA) (2011) by seven replicate analyses of chicken breast meat samples, were 1.0 μg/kg for AsB, 1.8 μg/kg for As^III^, 1.5 μg/kg for DMA^V^, 1.7 μg/kg for MMA^V^, and 1.2 μg/kg for Rox, measured as dry weight of chicken breast meat. We used three standard reference materials, SRM1640a (trace elements in natural water, obtained from the National Institute of Standards and Technology, Gaithersburg, MD), DORM-4 (fish muscle, obtained from the National Research Council of Canada, Ottawa, Canada), and BCR627 (tuna, obtained from the Institute for Reference Materials and Measurements, Belgium), for method development. Our results were in good agreement with the certified values (see Supplemental Material, “Quality Assurance”). Because there was no standard reference material for chicken meat certified for arsenic species, we prepared an in-house reference sample by adding 10 μg/L As standard mixture to a low-arsenic chicken breast meat sample purchased from a local food market. This reference sample was analyzed in triplicate along with each of the seven batches of chicken breast samples analyzed. The measured concentrations were AsB [mean ± SD, 11.1 ± 0.6 μg/L; coefficient of variation (CV) = 6%; *n* = 21], As^III^ (12 ± 1 μg/L; CV = 8%; *n* = 21), DMA^V^ (10 ± 1 μg/L; CV = 10%; *n* = 21), MMA^V^ (11 ± 1 μg/L; CV = 10%, *n* = 21), As^V^ (10 ± 1 μg/L; CV = 12%; *n* = 21), and Rox (11 ± 1 μg/L; CV = 11%; *n* = 21). During each batch of analysis, we also analyzed a solution containing 4.5 μg/L AsB, a stable arsenic species. The results (mean ± SD, 4.3 ± 0.2 μg/L; CV = 5.7%) indicated good reproducibility among the seven batches analyzed on separate days.

### Statistical Analysis

Statistical analyses were performed by using SPSS version 20.0 (IBM Corp, Armonk, NY). Arithmetic mean, standard deviation, and coefficient of variation of arsenic concentrations were calculated based on the results from duplicate analyses of multiple chicken samples in each test group. Sample size (*n*) in the tables and figures referred to the number of different chickens. They were each from one of the 16 pens that initially contained 100 chickens per pen.

We used two-way analysis of variance (ANOVA) to analyze the effect of Roxarsone treatment and age on the concentration of arsenic species over 35 days. We initially tested sex (male and female) and strains (Ross and Cobb) on the concentrations of arsenic species; however, their effects were not significant for any arsenic species. Therefore, we excluded sex and strain from the statistical model.

Mann–Whitney *U*-test was used to analyze the significance of difference between Rox-fed and control chickens on day 35. Spearman correlation test was performed to investigate the relationship between different arsenic species. Recognizing that most of the data for As^III^, Unknown, and Rox in the control group were below LOD, we conducted the sign test (SPSS, version 20.0; IBM Corp, Armonk, NY). for these three species (see Table S1) by comparing the range of their concentrations in the Rox-fed chickens to the LOD. The two-way ANOVA allowed us to assess on which day after the termination of Rox feeding the concentrations of arsenic species no longer significantly differed from the control treatment (see Table S2).

### Pharmacokinetic Analysis

The concentrations of arsenic species in chicken breast tissues were determined at each time point (day 28 to 35). The pharmacokinetic parameters, including elimination rate constant (*K*) and elimination half-life (*t_1/2_*), were determined by the compartmental method using Graphpad Prism 6 (GraphPad Software, San Diego, CA, USA). The formula for one-phase decay model is expressed as *Y* = (*Y_0_* – *Yt*) × exp(–*K* × *X*) + *Yt*, where *Y_0_* is the *Y* value when *X* (time) is zero; *Yt* is the *Y* value at infinite time or when *Y* value does not change significantly with time; *K* is the rate constant. Half-life is computed as ln(2)/*K*.

## Results

### Arsenic Species Found in Chicken Breasts

**Figure 1:**
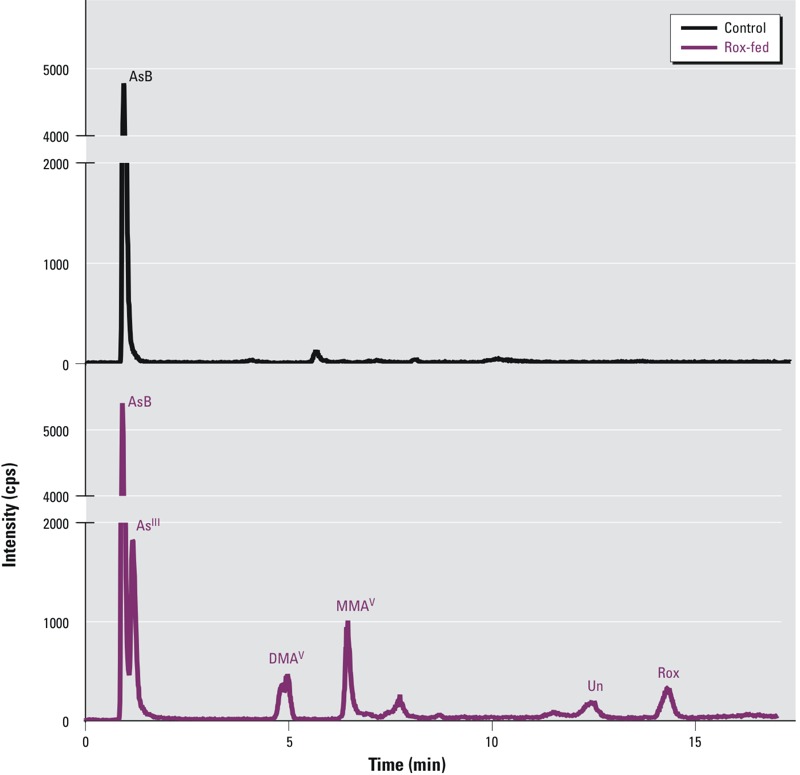
Chromatograms obtained from HPLC-ICPMS analyses of breast samples from a control chicken (top trace) and a Rox-fed chicken (bottom trace) collected on day 28 of the feeding experiment. The control chicken was given a basal diet not containing Roxarsone. The Rox-fed chicken was given a diet containing approximately 18 mg/kg Roxarsone during the first 28 days. Only arsenobetaine (AsB) was consistently present in the control chicken breast samples. AsB, arsenite (As^III^), dimethylarsinic acid (DMA^V^), monomethylarsonic acid (MMA^V^), Roxarsone, and an Unknown arsenic species (Un) were detected in the Rox-fed chicken breast samples.

Figure 1 shows typical chromatograms obtained from the analyses of a pair of chicken breast samples, one from the control group and the other from the Rox-fed group, both collected on day 28 of the feeding experiment. The chicken sample from the control group showed the presence of AsB as the major arsenic species (Figure 1, top trace). The chicken sample from the Rox-fed group showed the presence of detectable AsB, As^III^, DMA^V^, MMA^V^, Rox, and an unidentified arsenical (Unknown) (Figure 1, bottom trace).

Rox was not detectable in any of the samples from the 114 control chickens, but it was detected in all samples from the 115 Rox-fed chickens. Inorganic arsenite (As^III^) and methylated arsenicals (DMA^V^ and MMA^V^) were detected more frequently in the Rox-fed chicken samples than in the control chicken samples. As^III^, DMA^V^ and MMA^V^ were detected in 98% (113 samples), 93% (107), and 100% (115), respectively, of the Rox-fed chicken samples; they were detectable in 26% (22), 92% (106), and 92% (106) of the control chicken samples. The concentration of As^V^ in both the control and Rox-fed chickens was below LOD of 1.7 μg/kg. A possible explanation for the low concentration of As^V^ in the chicken breast could be that a substantial fraction of absorbed As^V^ was reduced to As^III^ (Vahter and Envall 1983; Vahter and Marafante 1985; Radabaugh and Aposhian 2000) before it was distributed in chicken breasts. A new arsenic species, whose chemical structure has yet to be identified, was detectable in 114 samples (99%) from the Rox-fed chickens. This new arsenic species was not detectable in any of the samples from the control chickens. Arsenobetaine (AsB) was detectable in all samples from both the control and Rox-fed chickens. Each of these arsenic species was quantified and the results from the analyses of 114 control chicken samples and 115 Rox-fed samples are summarized in Table 2.

**Table 2:** Concentrations (μg/kg) of individual arsenic species in the breast meat samples of 114 control chickens and 115 Rox-fed chickens over the 35-day feeding period.

Age	As^III^ in control		As^III ^in Rox-fed		Unknown^*a*^ in control		Unknown in Rox-fed		Rox in control		Rox in Rox-fed		*n* of control	*n* of Rox-fed
	Mean ± SD	CV	Mean ± SD	CV	Mean ± SD	CV	Mean ± SD	CV	Mean ± SD	CV	Mean ± SD	CV		
Day 0	ND	ND	ND	ND	ND	ND	ND	ND	ND	ND	ND	ND	8	8
Day 1	3.54 ± 1.10	31%	4.60 ± 2.27	49%	ND	ND	1.72 ± 0.61	35%	ND	ND	5.92 ± 1.92	32%	8	8
Day 2	1.27 ± 1.14	90%	11.54 ± 5.43	47%	ND	ND	4.68 ± 2.54	54%	ND	ND	9.44 ± 5.18	55%	6	6
Day 3	ND	ND	11.63 ± 2.95	25%	ND	ND	4.99 ± 1.51	30%	ND	ND	11.27 ± 1.93	17%	8	7
Day 4	ND	ND	21.59 ± 8.00	37%	ND	ND	6.04 ± 2.51	42%	ND	ND	12.11 ± 3.97	33%	8	8
Day 7	ND	ND	27.78 ± 7.39	27%	ND	ND	3.83 ± 1.06	28%	ND	ND	5.06 ± 1.06	21%	8	8
Day 14	ND	ND	10.67 ± 4.30	40%	ND	ND	2.33 ± 1.21	52%	ND	ND	2.77 ± 0.65	23%	7	8
Day 21	0.57 ± 0.22	39%	3.93 ± 0.93	24%	ND	ND	0.61 ± 0.25	41%	ND	ND	1.51 ± 0.32	21%	8	7
Day 28	ND	ND	30.11 ± 18.33	61%	ND	ND	5.03 ± 1.44	29%	ND	ND	5.14 ± 2.11	41%	8	8
Day 29	ND	ND	19.40 ± 3.46	18%	ND	ND	3.20 ± 0.33	10%	ND	ND	3.69 ± 0.70	19%	6	5
Day 30	ND	ND	14.95 ± 5.89	39%	ND	ND	2.16 ± 0.68	31%	ND	ND	1.62 ± 0.16	10%	6	7
Day 31	ND	ND	4.24 ± 0.38	9%	ND	ND	0.98 ± 0.28	29%	ND	ND	0.66 ± 0.22	33%	7	8
Day 32	ND	ND	2.89 ± 0.63	22%	ND	ND	0.63 ± 0.21	33%	ND	ND	0.69 ± 0.14	20%	5	7
Day 33	ND	ND	2.57 ± 1.25	49%	ND	ND	0.45 ± 0.13	29%	ND	ND	0.54 ± 0.21	39%	7	7
Day 34	ND	ND	2.47 ± 0.55	22%	ND	ND	0.73 ± 0.16	22%	ND	ND	0.48 ± 0.11	23%	6	5
Day 35	ND	ND	3.10 ± 1.61	52%	ND	ND	0.82 ± 0.29	35%	ND	ND	0.41 ± 0.04	10%	8	8

Age	AsB in control		AsB in Rox-fed		DMA^V^ in control		DMA^V^ in Rox-fed		MMA^V^ in control		MMA^V^ in Rox-fed		*n* of control	*n* of Rox-fed
	Mean ± SD	CV	Mean ± SD	CV	Mean ± SD	CV	Mean ± SD	CV	Mean ± SD	CV	Mean ± SD	CV		
Day 0	ND	ND	ND	ND	ND	ND	ND	ND	ND	ND	ND	ND	8	8
Day 1	5.58 ± 1.34	24%	5.37 ± 1.65	31%	1.43 ± 0.74	52%	1.92 ± 0.58	30%	0.52 ± 0.22	42%	1.25 ± 0.31	25%	8	8
Day 2	14.95 ± 7.41	50%	23.94 ± 10.24	43%	2.42 ± 0.53	22%	4.52 ± 1.12	25%	1.39 ± 0.16	12%	3.13 ± 0.48	15%	6	6
Day 3	27.68 ± 5.66	20%	33.18 ± 9.18	28%	2.99 ± 0.95	32%	4.62 ± 1.56	34%	1.44 ± 0.50	35%	2.40 ± 0.91	38%	8	7
Day 4	37.90 ± 12.67	33%	36.01 ± 7.28	20%	2.53 ± 0.41	16%	5.37 ± 1.59	30%	1.73 ± 0.53	31%	4.49 ± 1.58	35%	8	8
Day 7	22.80 ± 2.76	12%	27.22 ± 5.67	21%	2.26 ± 0.63	28%	3.69 ± 1.03	28%	3.50 ± 1.07	31%	5.99 ± 1.45	24%	8	8
Day 14	31.58 ± 6.08	19%	30.72 ± 4.40	14%	1.93 ± 0.26	13%	2.82 ± 1.16	41%	1.38 ± 0.39	28%	2.14 ± 0.19	9%	7	8
Day 21	17.57 ± 7.76	44%	14.34 ± 3.61	25%	1.89 ± 0.69	37%	2.37 ± 0.49	21%	1.17 ± 0.61	52%	1.93 ± 0.79	41%	8	7
Day 28	25.94 ± 8.07	31%	24.77 ± 5.42	22%	3.43 ± 1.97	57%	13.48 ± 11.47	85%	4.30 ± 1.97	46%	8.67 ± 3.77	43%	8	8
Day 29	37.99 ± 11.59	31%	30.93 ± 10.26	33%	2.69 ± 0.67	25%	11.96 ± 4.04	34%	2.43 ± 0.40	16%	6.07 ± 2.18	36%	6	5
Day 30	40.66 ± 11.42	28%	37.09 ± 16.88	46%	1.68 ± 0.65	39%	1.81 ± 0.35	19%	1.65 ± 0.44	27%	2.04 ± 0.45	22%	6	7
Day 31	21.68 ± 6.40	30%	18.61 ± 3.64	20%	1.29 ± 0.40	31%	0.90 ± 0.12	13%	0.79 ± 0.23	29%	0.85 ± 0.16	19%	7	8
Day 32	27.46 ± 9.17	33%	25.59 ± 9.11	36%	1.55 ± 0.21	14%	1.55 ± 0.50	32%	1.32 ± 0.23	17%	1.33 ± 0.44	33%	5	7
Day 33	25.55 ± 6.91	27%	24.48 ± 5.95	24%	0.75 ± 0.17	23%	1.18 ± 0.26	22%	0.69 ± 0.14	20%	1.01 ± 0.27	27%	7	7
Day 34	29.40 ± 12.49	42%	22.13 ± 6.30	28%	1.00 ± 1.06	106%	1.00 ± 0.74	74%	1.22 ± 0.49	40%	1.04 ± 0.29	28%	6	5
Day 35	30.99 ± 11.30	36%	33.50 ± 13.93	42%	1.32 ± 0.18	14%	1.80 ± 0.48	27%	1.14 ± 0.27	24%	1.42 ± 0.41	29%	8	8
Note: *n* is the number of chickens. ND is below the LOD of 1.0 μg/kg for AsB, 1.8 μg/kg for As^III^, 1.5 μg/kg for DMAv, 1.7 μg/kg for MMAv, 1.3 μg/kg for Unknown, and 1.2 μg/kg for Rox in the chicken breast meat samples in dry weight. Unknown is an arsenic species whose chemical structure is not yet identified. Mann–Whitney *U* tests were done for each pair containing one sample from the control group and one sample from the Rox-fed group of the same strain of chickens. Breasts samples were collected on day 35, 7 days after termination of Roxarsone feeding. *P*-value of significance is 0.05.														

### Comparison between the Control and Rox-Fed Chickens

Table 3 shows the results from the two-way ANOVA of each arsenic species present in more than 100 control chickens and more than 100 Rox-fed chickens. The comparison between the Rox-fed chickens and the control chickens in the concentrations of five arsenic species, including As^III^ (*p* ≤ 0.001), DMA^V^ (*p* ≤ 0.001), MMA^V^ (*p* = 0.01), Unknown (*p* ≤ 0.001), and Rox (*p* ≤ 0.001), showed significantly higher arsenic in the Rox-fed chickens than in the control chickens. The effect of age of chickens was significant for the concentrations of all six arsenic species (*p* ≤ 0.001). The effect of Roxarsone treatment changed significantly with age for the concentrations of all arsenic species (*p* ≤ 0.001) except AsB (*p* = 0.63).

**Table 3:** *p*-Values from two-way ANOVA comparing the concentrations of each arsenic species between the control and Rox-fed groups over the 35-day feeding period.

Source of variation	AsB	As^III^	DMA^V^	MMA^V^	Unknown	Rox
Treatment	0.76	< 0.001*	< 0.001*	< 0.001*	< 0.001*	< 0.001*
Age	< 0.001*	< 0.001*	< 0.001*	< 0.001*	< 0.001*	< 0.001*
Treatment × age	0.63	< 0.001*	< 0.001*	< 0.001*	< 0.001*	< 0.001*
*p-Value of significance is 0.05.						

AsB was the only species that had no significant difference (*p* = 0.76) in the concentration between the control chickens and the Rox-fed chickens. This result was understandable because the basal diet for all chickens contained approximately 0.03–0.1 μg/g AsB. The source of AsB was from fish that is commonly used as a protein source in chicken diets. In this study, AsB was present at similar concentrations in the food to both the control group and Rox-fed group of chickens. Therefore, AsB was an appropriate internal standard.

### Temporal Profiles of Each Arsenic Species

From the speciation analyses of 229 chicken samples collected on different days over the 35-day feeding experiment, we were able to obtain temporal profiles for individual arsenic species. Because each group of chickens was exposed to the same feed and because AsB was not metabolized, we normalized the concentrations of individual arsenic species in each chicken against the concentration of AsB in the respective chicken. With AsB as an internal standard, this normalization minimizes potential analytical fluctuations. Data without normalization against AsB is shown in Figure S1.

**Figure 2:**
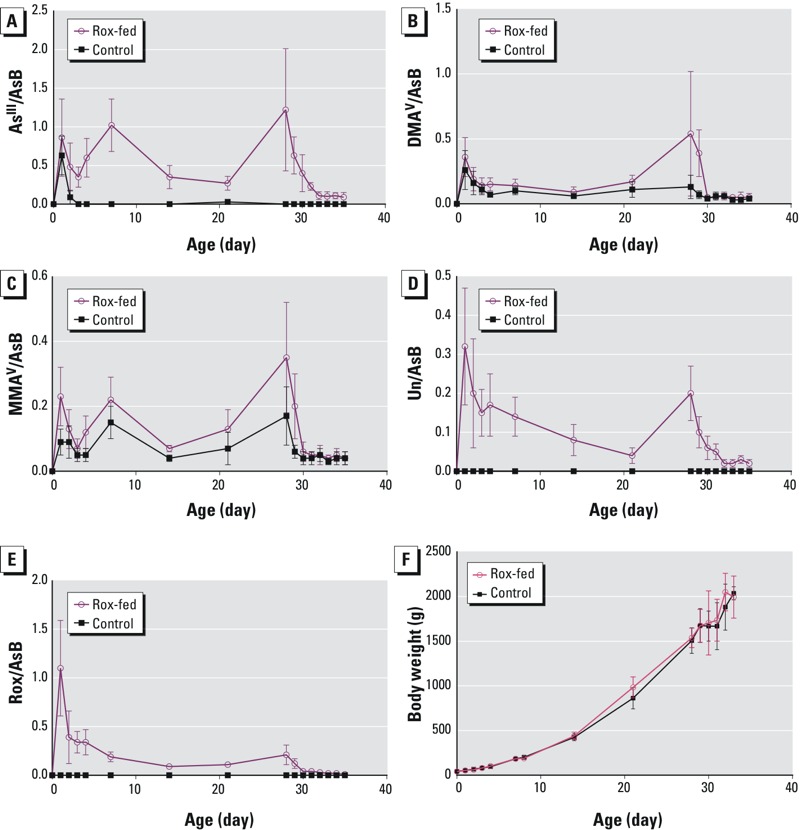
Concentrations of (*A*) As^III^, (*B*) DMA^V^, (*C*) MMA^V^, (*D*) Unknown arsenic species (Un), and (*E*) Rox, normalized against AsB, in the breast samples of control chickens and Rox-fed chickens over the entire 35-day feeding period. (*F*) Body weight of chickens over the 35-day feeding experiment. Data represent mean values and error bars represent one standard deviation from duplicate analyses of each of 5–8 chicken samples.

**Figure 3:**
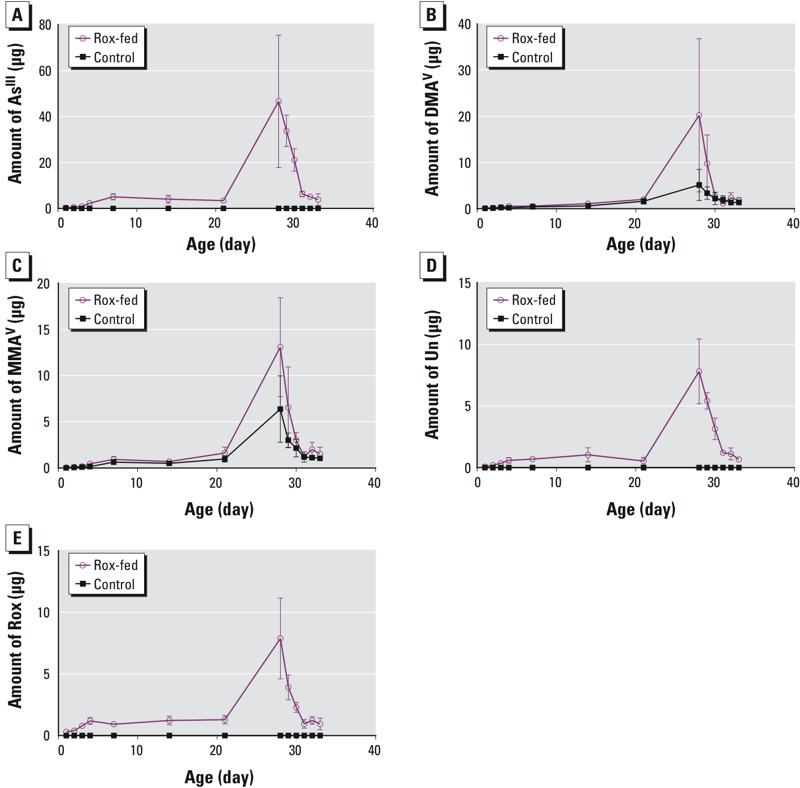
Content of (*A*) As^III^, (*B*) DMA^V^, (*C*) MMA^V^, (*D*) Unknown arsenic species (Un), and (*E*) Rox in the breast samples of control and Rox-fed chickens. The amount of arsenic species (μg) was obtained by multiplying the concentrations of arsenic species in each sample by its sample-specific body weight. Data represent mean values and error bars represent one standard deviation from duplicate analyses of 5–8 chicken samples.

Figure 2 shows that the concentrations of As^III^ (Figure 2A), DMA^V^ (Figure 2B), MMA^V^ (Figure 2C), and Unknown (Figure 2D) in the Rox-fed chickens increased in a similar trend to that of Rox (Figure 2E) during the first 28 days when these chickens were fed the Rox-containing diet. Their concentrations all reached maximum on day 28, the last day that Rox was fed. The rapid decreases in arsenic concentrations from day 28 to day 35 reflected elimination of arsenic from the chickens during the Rox withdrawal period. The elimination kinetics will be discussed later. The apparent lower concentrations of arsenic species between day 7 and day 21 could be due to rapid growth of chickens, resulting in distribution of arsenic species in larger masses of chicken breasts. Indeed, Figure 2F shows rapid body weight gains of both groups of chickens in this period. Taking into account of the chicken growth (and body weight), we multiplied the concentration of each arsenic species by the sample-specific body weight. Figure 3 shows continual increases of As^III^ (Figure 3A), DMA^V^ (Figure 3B), MMA^V^ (Figure 3C), the Unknown arsenic species (Figure 3D), and Rox (Figure 3E) in the Rox-fed chickens in the first 28 days. The average amount of arsenic species in the chickens fed 28 days of Rox were 38 ± 19 μg As^III^, 20 ± 16 μg DMA^V^, 13 ± 5 μg MMA^V^, 8 ± 3 μg Rox, and 8 ± 3 μg Unknown arsenic species.

### Elimination of Arsenic Species

**Figure 4:**
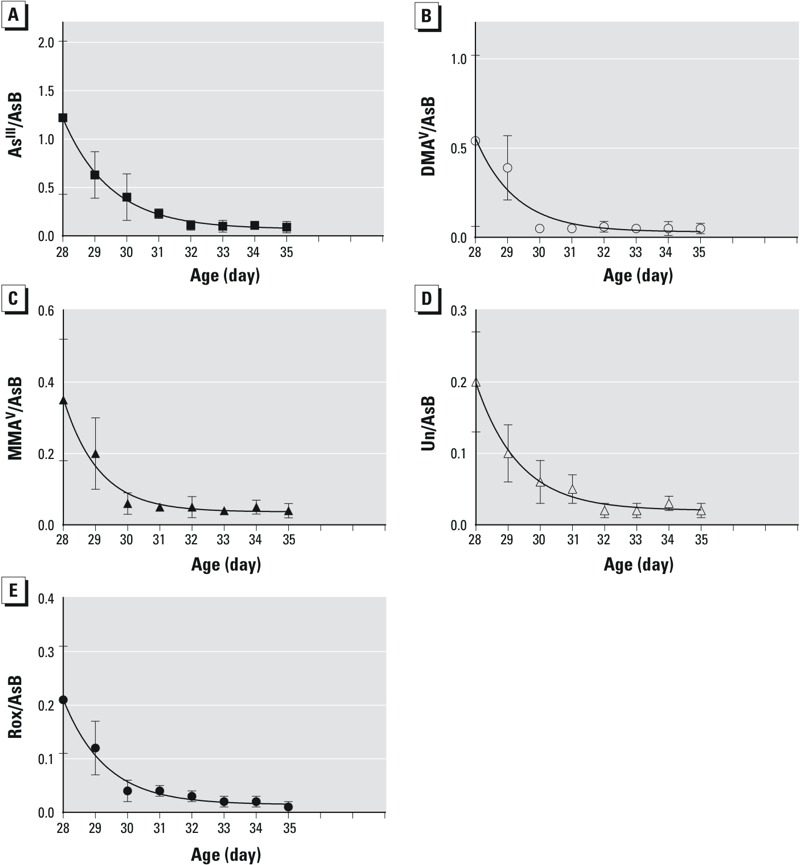
Concentrations of (*A*) As^III^, (*B*) DMA^V^, (*C*) MMA^V^, (*D*) Unknown arsenic species, and (*E*) Rox normalized against AsB, in the breast samples of Rox-fed chicken. Eight Rox-fed samples were collected each day from day 28 to day 35. Day 28 was the last day when these chickens were fed Roxarsone. From day 29 to day 35, all chickens were fed the control food that did not contain Roxarsone. Data points were presented as mean and one standard deviation from duplicate analyses of each of the 5–8 breast samples. The curve represents the best fit of the data using one-phase exponential decay function.

Figure 4 summarizes elimination of As^III^ (Figure 4A), DMA^V^ (Figure 4B), MMA^V^ (Figure 4C), the Unknown arsenic species (Figure 4D), and Rox (Figure 4E) individual arsenic species from the Rox-fed chicken breasts after the feeding of Rox stopped on day 28. These results show patterns of decreasing arsenic concentrations in the chicken breast from day 28 to day 35. Fitting the concentrations of arsenic species on each day after the termination of Rox feeding with a one-phase exponential decay model enabled us to estimate the elimination kinetics and half-life of individual arsenic species. As shown in Table 4, the half-lives for all arsenic species are < 1 day. As^III^ has the longest retention in chicken breast (*t_1/2_* = 1 day) and DMA^V^ has the shortest retention (*t_1/2_* = 0.4 day). The other three arsenic species, Rox, MMA^V^ and the new metabolite had a similar half-life (*t_1/2_* = 0.7 day).

**Table 4:** The elimination rate constant (*K*), elimination half-life (*t_1/2_*), *Y_0_* and *Y_t_* for individual arsenic species in the one-phase decay elimination model.

Model parameter	As^III^	DMA^V^	MMA^V^	Unknown	Rox
*K* (day^–1^)	0.69	1.90	0.90	0.93	0.99
*t*_*1/2*_ (day)	1.00	0.37	0.73	0.74	0.70
(95% CI)	(0.70, 1.80)	(0.28, 0.58)	(0.50, 1.35)	(0.54, 1.20)	(0.52, 1.11)
*Y*_*0*_	2.38	4.86	0.82	0.51	0.56
*Y*_*t*_	0.06	0.02	0.04	0.02	0.02

Figure 4 also shows that after several days of elimination, the concentrations of arsenic species appears to have no significant further decrease. We conducted two-way ANOVA on the arsenic concentration data from day 28 through to day 35. We found that for the faster eliminating species DMA^V^ and MMA^V^, starting on day 30 their concentrations did not significantly differ from the final concentrations on day 35. The *p*-value for comparison between day 29 (or day 28) and day 35 were < 0.01, while the *p*-value for comparison between day 30 (or age > day 30) and day 35 were > 0.76 for DMA^V^ and MMA^V^. For As^III^, Unknown, and Rox, starting on day 31, the concentrations did not significantly differ from their concentrations on day 35. The *p*-value for comparison between day 30 (or age < day 30) and day 35 were < 0.02, while the *p*-value for comparison between day 31 (or age > day 31) and day 35 were > 0.14 for As^III^, Unknown, and Rox.

### Residual Arsenic Species after Termination of Rox Feeding

**Figure 5:**
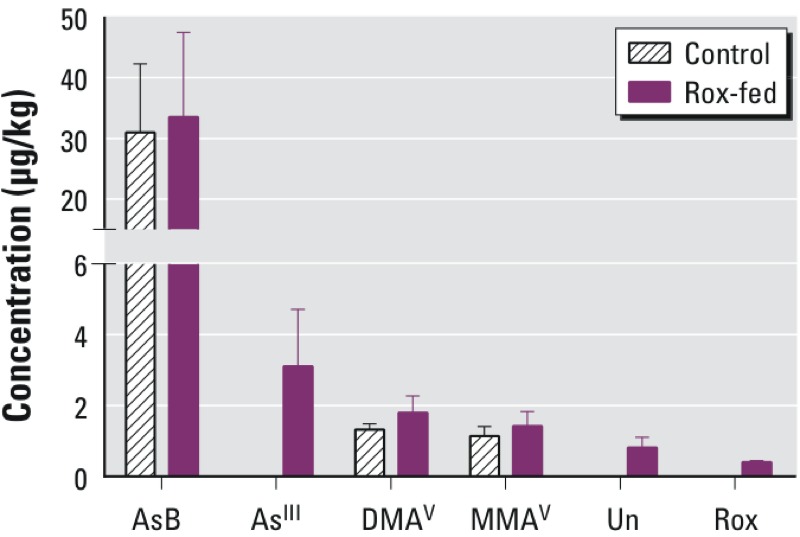
The mean concentrations of AsB, As^III^, DMA^V^, MMA^V^, Unknown arsenic species (Un), and Rox in eight control chickens and eight Rox-fed chickens on Day 35 (final day) of the feeding experiment. This was 7 days after the final feeding of Roxarsone on day 28. Error bars represent standard deviation from four replicate measurements of each of the eight chicken samples. The concentrations of As^III^, Rox, and Unknown are significantly higher (*p* < 0.01) in the Rox-fed chickens than in the control chickens. The concentrations of AsB are not significantly different (*p* > 0.01) between the control and the Rox-fed chickens.

Although Figure 4 shows rapid clearance of arsenic species, it was not clear whether the residual arsenic remaining in chicken breast was significantly different when comparing the control and the Rox-fed chickens. Therefore, we compared arsenic concentrations in eight control chickens and eight Rox-fed chickens on the last day. Figure 5 shows the concentrations of arsenic species in the control and Rox-fed chickens on day 35. The results of Mann Whitney *U* tests are shown in Table 5. Except for AsB (*p* = 0.88) and MMA^V^ (*p* = 0.13), As^III^ (*p* = 0.01), DMA^V^ (*p* = 0.02), Unknown (*p* < 0.001), and Rox (*p* < 0.001) in the Rox-fed group were significantly higher than those in the control group.

**Table 5:** Mann–Whitney *U* tests comparing the concentrations of individual arsenic species in the breast samples between the eight control chickens and the eight Rox-fed chickens on day 35.

Arsenic species	Control (μg/kg) (mean ± SD)	Rox-fed (μg/kg) (mean ± SD)	*p*-Value
AsB	31 ± 11	34 ± 14	0.88
As^III^	ND	3.1 ± 1.6	0.01*
DMA^V^	1.3 ± 0.2	1.8 ± 0.5	0.02*
MMA^V^	1.1 ± 0.3	1.4 ± 0.4	0.13
Unknown	ND	0.82 ± 0.29	< 0.001*
Rox	ND	0.41 ± 0.04	< 0.001*
Note: ND is below the LOD of 1.0 μg/kg for AsB, 1.8 μg/kg for As^III^, 1.5 μg/kg for DMAv, 1.7 μg/kg for MMAv, 1.3 μg/kg for Unknown, and 1.2 μg/kg for Rox in the chicken breast meat samples in dry weight. Unknown is an arsenic species whose chemical structure is not yet identified. Mann–Whitney *U* tests were done for each pair containing one sample from the control group and one sample from the Rox-fed group of the same strain of chickens. Breasts samples were collected on day 35, 7 days after termination of Roxarsone feeding. *p*-Value of significance is 0.05.			

The concentrations of residual As^III^ in Rox-fed chicken were from 0.41 to 3.1 μg/kg in chicken breasts (Figure 5 and Table 5). The concentrations of As^III^, Rox, DMA^V^, MMA^V^, and Unknown were an order of magnitude lower than the concentrations of AsB (31 ± 11 μg/kg in the control chickens and 34 ± 14 μg/kg in the Rox-fed chickens).

### Correlation between Arsenic Species

Rox showed significant correlation with As^III^ (*r* = 0.74, *p* < 0.001), DMA^V^ (*r* = 0.80, *p* < 0.001), MMA^V^ (*r* = 0.71, *p* < 0.001), and Unknown (*r* = 0.87, *p* < 0.001). Especially for the Unknown arsenic species, such a strong correlation with Rox suggests it might be a direct metabolite of Rox.

## Discussion

This study extensively determined the concentrations of individual arsenic species in chicken breast meat samples from 229 chickens, of which 115 were fed a Rox-containing diet and 114 were controls (Table 2). During the 28 days when chickens were given a Rox-containing food, the concentrations of As^III^, Rox, DMA^V^, MMA^V^, and a new arsenic species (Unknown) in breast muscle increased to a maximum on day 28 (Figures 2 and 3). The concentrations of these arsenic species were significantly higher in the Rox-fed chickens than in the control chickens (*p* ≤ 0.001).

Starting on day 29, all chickens were fed the diet containing no Rox. By day 35, the Rox-fed chickens had 7 days to excrete arsenic from the body. The poultry industry standard regulated by the U.S. FDA (2015a) is to have a 5-day clearance period. Our results show that the majority of arsenic species was excreted rapidly, with half-lives ranging from 0.4 day for DMA^V^ to 0.7 day for MMA^V^, Rox and Unknown arsenic species, and 1 day for As^III^. Trivalent arsenicals readily interact with cysteine groups in proteins (Shen et al. 2013), such as tubulin and myosin (Menzel et al. 1999); these interactions could contribute to the longer retention of As^III^ in chicken breasts. Adding papain enhanced the extraction of As^III^ from chicken breasts (see Figure S2), which also suggested that As^III^ could be present in bound form. After 5 days following the withdrawal of Rox from the feed, there was no further significant decrease of arsenic concentrations in chicken breast meat. Thus, a 5-day clearance period seems reasonable. However, after the 7-day withdrawal period, the concentrations of four arsenic species, As^III^, DMA^V^, Rox, and the Unknown, were significantly higher in the Rox-fed chickens than in the control chickens (Table 5). The arsenic species in the chicken breasts were not completely cleared to the background level of the control.

In previous studies, Morrison (1969) and Brugman et al. (1967) pointed out that feeding chicken or lamb on chicken litter containing Roxarsone did not cause arsenic residues to accumulate in the edible tissues. However, the authors also mentioned that the amount of litter consumed was not large enough to lead to any detectable increase of arsenic. Nachman et al. (2013) detected the concentrations of inorganic arsenicals (arsenite and arsenate together) in conventional supermarket chicken meat samples and found the concentrations in Rox-positive samples had geometric mean (GM) of 2.3 μg/kg (95% CI: 1.7, 3.1). The concentration of Rox in Rox-positive samples had GM of 1.3 μg/kg (95% CI: 1.0, 1.7). In our study, the overall concentrations of arsenic species in the chicken breast meat after 7-day withdrawal period were similar to those reported by Nachman et al. (2013). The concentration of Rox (0.41 ± 0.04 μg/kg) on day 35 was slightly lower than the results of Nachman et al. (2013) and the concentration of As^III^ (3.1 ± 1.6 μg/kg) was slightly higher. In addition to the determination of As^III^ and Rox in the chicken breast meat, we also detected MMA^V^ (1.4 ± 0.4 μg/kg), DMA^V^ (1.8 ± 0.5 μg/kg), and a new arsenic metabolite (0.8 ± 0.3 μg/kg) whose chemical structure has yet to be identified.

Using the concentrations of arsenic species, we determined in the chicken breast meat after the 7-day withdrawal period, we could estimate the human daily intake of arsenic from the consumption of these Rox-fed chicken. The residual concentration of As^III^ in Rox-fed chicken was 3.1 ± 1.6 μg/kg. For an average consumption of 98 g chicken per day (USDA 2014), the average daily intake of As^III^ from eating this chicken would be 0.3 ± 0.2 μg/day. The summed concentrations of all arsenic metabolites (excluding the non-toxic arsenobetaine) in Rox-fed chicken samples after 7-day withdrawal was 7.6 μg/kg. From an average consumption of 98 g chicken meat per day, the average daily intake of all arsenic metabolites from chicken breast meat would be 0.7 μg/day or 0.01 μg/(day kg body weight) for a 70-kg adult. This is much lower than the World Health Organization (WHO 2011) provisional tolerable daily intake value of 3 μg/(day kg body weight) for inorganic arsenic. As a comparison, the upper limit of arsenic in drinking water is 10 μg/L (WHO 2008). The daily intake of arsenic from 2 L of water containing 10 μg/L arsenic would be 20 μg/day, or 0.3 μg/(day kg) for 70-kg adults. Water and food are the primary sources of human exposure to arsenic (Hughes et al. 2011; Kile et al. 2007; Newbigging et al. 2015; Schoof et al. 1999; Tao and Bolger 1999; Williams et al. 2005; WHO 2011). Trace concentrations of arsenic are present in all food items as arsenic is naturally occurring in the environment. Although the contribution of arsenic from chicken breast meat is low, it is important to minimize exposure to arsenic from all possible sources.

## Conclusions

The present study provides information on the concentrations of individual arsenic species in chicken breast throughout the 35-day feeding period. Feeding Roxarsone to broiler chickens increased the concentrations of As^III^, Rox, and a new arsenic metabolite in chicken breast meat. Although arsenic species were excreted rapidly from the chickens during the Rox withdrawal period, the residual arsenic concentrations in chicken breast meat 7 days after terminating Rox feeding remained significantly higher in the Rox-fed chickens than in the control chickens. However, our estimates suggest that adults consuming a moderate amount of chicken breast meat would not exceed the WHO provisional tolerable daily arsenic intake level given residual arsenic concentrations consistent with those in our Rox-fed study sample.

## Supplemental Material

(680 KB) PDF
