# Supplementary material for: Arsenic Species in Chicken Breast: Temporal Variations of Metabolites, Elimination Kinetics, and Residual Concentrations
Source: Environ Health Perspect. 2016 Mar 18;124(8):1174–81. doi: 10.1289/ehp.1510530 (PMC4977061; doi:10.1289/ehp.1510530)
Supplement: (680 KB) PDF [file ehp.1510530.s001.acco.pdf]

Environ Health Perspect
DOI: 10.1289/ehp.1510530

Note to readers with disabilities: *EHP* strives to ensure that all journal content is accessible to all readers. However, some figures and Supplemental Material published in *EHP* articles may not conform to 508 standards due to the complexity of the information being presented. If you need assistance accessing journal content, please contact ehp508@niehs.nih.gov. Our staff will work with you to assess and meet your accessibility needs within 3 working days.

Supplemental Material

Arsenic Species in Chicken Breast: Temporal Variations of Metabolites, Elimination Kinetics, and Residual Concentrations

Qingqing Liu, Hanyong Peng, Xiufen Lu, Martin J. Zuidhof, Xing-Fang Li, and X. Chris Le

Table of Contents

Analytical Procedures
- Reagents and Standards
- Instrumentation
- Sample preparation
- Enzyme-assisted extraction of arsenic species
- Determination of arsenic species using HPLC-ICPMS
- Determination of total arsenic after acid digestion of chicken samples
- Extraction Efficiency
- Column Recovery
- Limit of Detection (LOD)
- Quality Assurance

Table S1. Sign test comparing As^{III}, Unknown arsenic species, and Rox between the control and Rox-treated groups over the 35-day feeding period.

Table S2. P values from two-way ANOVA comparing the concentration of each arsenic species in the last 7 days of the 35-day feeding study. The concentration of each arsenic species on day 35 was used as the reference for comparison with the other days (age).

Figure S1. Concentrations of As^{III} (a), DMA^{V} (b), MMA^{V} (c), Unknown arsenic species (d), and Rox (e), without normalization against AsB, in the breast samples of control and Rox-fed chickens over the

35-day feeding period. Data represent mean values and error bars represent one standard deviation from replicate analyses of each of 5-8 chicken samples.

Figure S2. Chromatograms obtained from HPLC-ICPMS analyses of a chicken meat sample after different extraction methods. The peaks labeled with numbers 1 through to 6 correspond to AsB, As^{III}, DMA^{V}, MMA^{V}, Unknown and Rox, respectively. The extraction methods were with water-methanol (left panel) and with papain (right panel). (Liu et al. 2015).

References

Analytical Procedures

Reagents and Standards

Solutions of arsenobetaine (AsB), arsenite (As^{III}), arsenate (As^{V}), monomethylarsonic acid (MMA^{V}), dimethylarsinic acid (DMA^{V}), N-acetyl-4-hydroxy-m-arsanilic acid (NAHAA), and 3-nitro-4-hydroxyphenylarsonic acid (Rox) were prepared from arsenobetaine (98% purity, Tri Chemical Laboratories Inc., Japan), sodium m-arsenite (97.0%, Sigma, St. Louis, MO), sodium arsenate (99.4%, Sigma), monosodium acid methane arsonate (99.0%, Chem Service, West Chester, PA), cacodylic acid (98%, Sigma), N-acetyl-4-hydroxy-m-arsanilic acid (Pfaltz and Bauer Inc.), and 3-nitro-4-hydroxyphenylarsonic acid (98.1%, Sigma-Aldrich, St. Louis, MO), respectively, in deionized water (Milli-Q18.2 MΩ·cm, Millipore Corporation, Billerica. MA). HPLC grade methanol (Fisher Scientific, Fair Lawn, NJ) was used as solvents. Papain and sodium bicarbonate was purchased from Sigma.

A primary arsenic standard (Agilent Technologies, Santa Clara, CA) was used to prepare the calibration standard solutions for total arsenic determination. Three standard reference materials (SRM) were used. SRM1640a (trace elements in natural water) was obtained from National Institute of Standards and Technology (Gaithersburg, MD). It contains inorganic arsenate and arsenite, and the certified value is 8.075±0.070 µg/L for total arsenic. DORM-4 (fish protein certified reference material for trace metals) was obtained from National Research Council of Canada (Ottawa, ON, Canada). The certified value (6.80±0.64 mg/kg) is for total arsenic concentration. BCR627 (tuna fish meat) was obtained from Institute for Reference Materials and Measurements (IRMM), Belgium. It has certified concentrations of arsenobetaine (52±3 µmol/kg) and dimethylarsinic acid (2.0±0.3 µmol/kg).

The concentration of each arsenic species in the stock solution was 10 mg/L. The concentrations of these arsenic species in stock solutions were calibrated against the primary arsenic standard. Calibration solutions of arsenic species (0.1, 0.5, 1, 5, 10 μg/L) were freshly prepared by serial dilutions from the stock solutions before each batch of speciation analysis.

Instrumentation

An Agilent 1100 series high performance liquid chromatography (HPLC) system (Agilent Technologies, Germany), installed with a PRP-X110S anion exchange column (7 μm particle size, 150×4.1 mm; Hamilton, Reno, NV), was used for separation of arsenic species. An Agilent 7500cs inductively coupled plasma mass spectrometry (ICPMS) system (Agilent Technologies, Japan) and an AB SCIEX 5500 QTRAP electrospray mass spectrometry (ESIMS) system (Concord, ON, Canada) were used for detection of arsenic.

For quantification of arsenic species present in the chicken samples, the eluent from HPLC column was directly introduced into ICPMS at a flow rate of 2 mL/min. For identification of arsenic species, the eluent from the HPLC column was split so that 80% of the flow (1.6 mL/min) was introduced to ICPMS and 20% of the flow (0.4 mL/min) was introduced to ESIMS (Peng et al. 2014). This split was achieved by using a 300 series stainless steel tee (Valco Canada, Brockville, ON, Canada).

Sample preparation

Each chicken breast meat sample was homogenized separately in a blender. Then 10g of the homogenized samples were freeze-dried in a freeze dryer (FTS Systems, Stone Ridge, NY, USA). The freeze-dried samples were stored as crumbled powder in a -20 ^{o}C freezer. Weight of each sample was recorded before and after freeze-drying. The ratio of dry weight over

wet weight of each sample was used to convert the arsenic concentrations that were measured in freeze-dried sample to their concentrations in wet weight. On average, the ratio of dry weight over wet weight was 0.24±0.04.

Enzyme-assisted extraction of arsenic species

Arsenic species in the freeze-dried samples were extracted using an enzyme-assisted extraction method (Liu et al. 2015) with a slight modification. A freeze-dried sample (approximately 0.5 g weighed with a precision of 0.1 mg) and 50 mg papain were added to 10 mL deionized water. The mixture was sonicated at 15% amplitude and 20 KHz for 2 min, followed by a stop for 1 min, and further sonication for another 2 min. The mixture of the sample and papain in deionized water was then incubated in a 65-^{o}C water bath for 4 hours. After incubation, the temperature of the water bath was increased to 95°C to denature papain and stop its activity. Then the extracts were centrifuged at 4000g for 15 min. The supernatant was filtered through a 0.45-μm membrane and the filtrate was analyzed for arsenic speciation using HPLC-ICPMS.

Determination of arsenic species using HPLC-ICPMS

HPLC-ICPMS analysis of arsenic species was according to the method of Liu et al. (2015). "An anion exchange column was used along with two mobile phases and a gradient elution program. Mobile phase A contained 5% methanol and 95% deionized water. Mobile phase B contained 5% methanol and 60 mM NH_4HCO_3 in deionized water, pH 8.75. The gradient program started with 100% mobile phase A and 0% mobile phase B. Mobile phase B was linearly increased to 40% during the first 10 min, with corresponding decrease of mobile phase A to 60%. From 10 min to 17 min, mobile phase B continued to increase linearly to 100%. From 17 min to 18 min, mobile phase B returned to 0% and mobile phase A increased to

100%. 100% mobile phase A remained to the end of the chromatographic run (22 min). The flow rate was 2 mL/min. ICPMS provided element specific detection of arsenic at m/z 75. The peak areas of each arsenic species in the chromatograms obtained from HPLC-ICPMS analysis were used for the quantification of the concentrations of arsenic species".

Determination of total arsenic after acid digestion of chicken samples

The method of acid digestion was modified from the US EPA method 3050B (US EPA 1996). Briefly, a freeze-dried powder sample (0.3 g) was weighed into a 50-mL beaker, to which 25 mL concentrated nitric acid (HNO_3) was slowly added. The beaker was covered with a watch glass and left in a fume hood overnight. In the following morning, the beaker was placed on a hot plate that was heated to 200 ^{o}C. Digestion was complete when the solution became transparent and it was yellowish in color. The watch glass was then removed to allow for evaporation of the acid from the beaker until about 0.5 mL solution remaining. The residual solution was quantitatively transferred to a 15-mL tube and diluted to 5 mL with deionized water. The solution was either diluted with deionized water by another 10 times or directly analyzed for total arsenic using ICPMS. For quality assurance, standard reference material DORM-4 (fish muscle) was digested in the same manner and analyzed using ICPMS. Standard reference material SRM1640a was also used for quality assurance.

For determination of total arsenic in extracts, each extract was diluted by 10 times and the diluted solution was divided into 3 aliquots. SRM 1640a was added to two aliquots, making these aliquots to contain additional 5 μg/L and 10 μg/L arsenic, respectively. Total arsenic concentration in the extract was determined using ICPMS and the standard addition method.

Extraction Efficiency

A comparison between the concentration of arsenic in the extract and the total arsenic concentration provided information on the extraction efficiency. Five chicken breast meat samples, collected from day 7, day 14, day 21, day 28, and day 35 of the feeding experiment, were used to evaluate the extraction efficiency. These five chicken samples were chosen to represent the 5-week feeding experiment. They were from one pen that initially housed 100 chickens; and these five chickens were randomly selected and euthanized on day 7, day 14, day 21, day 28, and day 35, respectively. The different ages of the chickens correspond to their durations of exposure to Roxarsone. Therefore, the concentrations of arsenic species in these chickens were representative of the actual arsenic concentrations in the rest of chicken samples. Our results on the extraction efficiencies of arsenic in these representative samples ranged from 83% to 95%, with an average of (88±4)% (mean ± SD).

We also compared the sum of arsenic species obtained from the HPLC-ICPMS analyses of the extract and the total arsenic concentrations obtained from the direct ICPMS analyses of the acid-digested samples. Our results showed that the sum of arsenic species as a ratio of the total arsenic concentration was (80±17)% (mean ± SD, n=5).

Column Recovery

We evaluated the column recovery of arsenic species using the extracts of the five selected samples, representing day 7, 14, 21, 28 and 35 of the feeding study. Column recovery was assessed as the ratio of the concentrations of arsenic species determined with and without HPLC separation. It represented the sum of arsenic species determined using HPLC-ICPMS and the total arsenic concentration in the same extract determined using direct ICPMS analysis. Our tests from the five samples showed that the column recovery was (89±14)% (mean ± SD).

Limit of Detection (LOD)

The detection limits (LOD) were determined using the method of US EPA (2015). From the local food market in Edmonton, Canada, we purchased chicken breast meat to serve as blank samples. We spiked a mixture of arsenic standard (0.2 μg/L) to the chicken sample and then carried out seven replicate analyses of the samples. Standard deviations from the seven replicate analyses for each arsenic species, multiplied by the student's t value of 3.143 (for n=7), in combination with calibration of each arsenic species, gave rise to limits of detection. Calibration standards included concentrations of each arsenic species at 0.1, 0.5, 1, 5, and 10 μg/L. To assess the detection limit of the Unknown arsenic species, we used N-acetyl-4-hydroxy-m-arsanilic acid (NAHAA) as a surrogate because NAHAA had a similar retention time and similar peak shape as the Unknown arsenic species. The LOD for the seven arsenic species were 1.0 μg/kg for AsB, 1.8 μg/kg for As^{III}, 1.5 μg/kg for DMA^{V}, 1.7 μg/kg for MMA^{V}, 1.7 μg/kg for As^{V}, 1.3 μg/kg for NAHAA (surrogate for the Unknown arsenic species), and 1.2 μg/kg for Rox, measured in dry weight of chicken breast meat. We have determined that the ratio of the dry weight over the wet weight of chicken breast meat was 0.24±0.04. Thus the above detection limit can be converted to corresponding values in wet weight of chicken breast meat.

Quality Assurance

We used three standard reference materials for method development purpose. We used SRM1640a (trace elements in natural water) to assess the calibration. We used DORM-4 (fish protein certified reference material for trace metals) to assess acid digestion and the determination of total arsenic. We also determined concentrations of arsenic species in standard reference material BCR-CRM627 (tuna fish muscle tissue, from the Institute for Reference Materials and Measurements, Belgium). This reference material has certified values for

arsenobetaine (52±3 μmol/kg), dimethylarsinic acid (2.0±0.3 μmol/kg), and total arsenic concentration (4.8±0.3 mg/kg). Our results from 7 replicate analyses of BCR-CRM627 showed that the concentrations of arsenobetaine (51±2 μmol/kg), dimethylarsinic acid (2.2±0.1 μmol/kg), and total arsenic concentration (4.8±0.2 mg/kg) were in good agreement with the certified values. Because there is no chicken meat standard reference material certified for arsenic species, we prepared an in-house reference sample by adding 10 μg/L As standard mixture to a low-arsenic chicken breast meat sample purchased from the local food market. This reference sample was analyzed in triplicates along with every batch of chicken breast samples. Thus, from the total of 21 analyses, the measured concentrations of arsenic species were AsB (mean ± SD, 11.1 ± 0.6 μg /L; coefficient of variation (CV)=6%; n=21), As^{III} (12 ± 1 μg /L; CV= 8%; n=21), DMA^{V} (10 ± 1 μg /L; CV= 10%; n=21), MMA^{V} (11 ± 1 μg /L; CV= 10%, n=21), As^{V} (10 ± 1 μg /L; CV= 12%; n=21), and Rox (11 ± 1 μg /L; CV= 11%; n=21).

During each batch of sample analysis, we also analyzed a solution containing 4.5 μg/L AsB. From the seven batches of analyses on separate days, the results showed good agreement (mean ± SD, 4.3 ± 0.2 μg/L; CV=5.7%). These results indicated good reproducibility between days. In addition, a standard mixture (1 μg/L of As) was re-analyzed between every 10 samples. Calibration solutions were re-run after every 20 samples.

Table S1. Sign test comparing As^{III}, Unknown arsenic species, and Rox between the control and Rox-treated groups over the 35-day feeding period.

As species	Control (μg/kg)		Rox-fed (μg/kg)		P value [a]
	Min-Max	Median	Min-Max	Median	
As^{III}	0.36-0.36	0.36	N.D.[a] -70.6	6.54	<0.001*
Unknown	0.26-0.26	0.26	N.D-9.91	1.45	<0.001*
Rox	0.24-0.24	0.24	N.D.-18.6	1.90	<0.001*

* P-value is significant at the 0.05 level.
[a] N.D.: below detection limit of 1.0 μg/kg for AsB, 1.8 μg/kg for As^{III}, 1.5 μg/kg for DMA^{V}, 1.7 μg/kg for MMA^{V}, 1.3 μg/kg for Unknown, and 1.2 μg/kg for Rox in the chicken breast meat samples in dry weight.

Table S2. P values from two-way ANOVA comparing the concentration of each arsenic species in the last 7 days of the 35-day feeding study. The concentration of each arsenic species on day 35 was used as the reference for comparison with the other days (age).

Age	As^{III}	DMA^{V}	MMA^{V}	Unknown	Rox
28	<0.001*	<0.001*	<0.001*	<0.001*	<0.001*
29	<0.001*	<0.001*	0.007*	<0.001*	<0.001*
30	0.002*	0.94	0.76	0.02*	0.01*
31	0.27	0.88	0.91	0.14	0.33
32	0.93	0.91	0.80	0.86	0.29
33	0.81	0.90	0.93	0.56	0.66
34	0.92	0.87	0.97	0.72	0.61

*. The difference of mean is significant at 0.05 level.

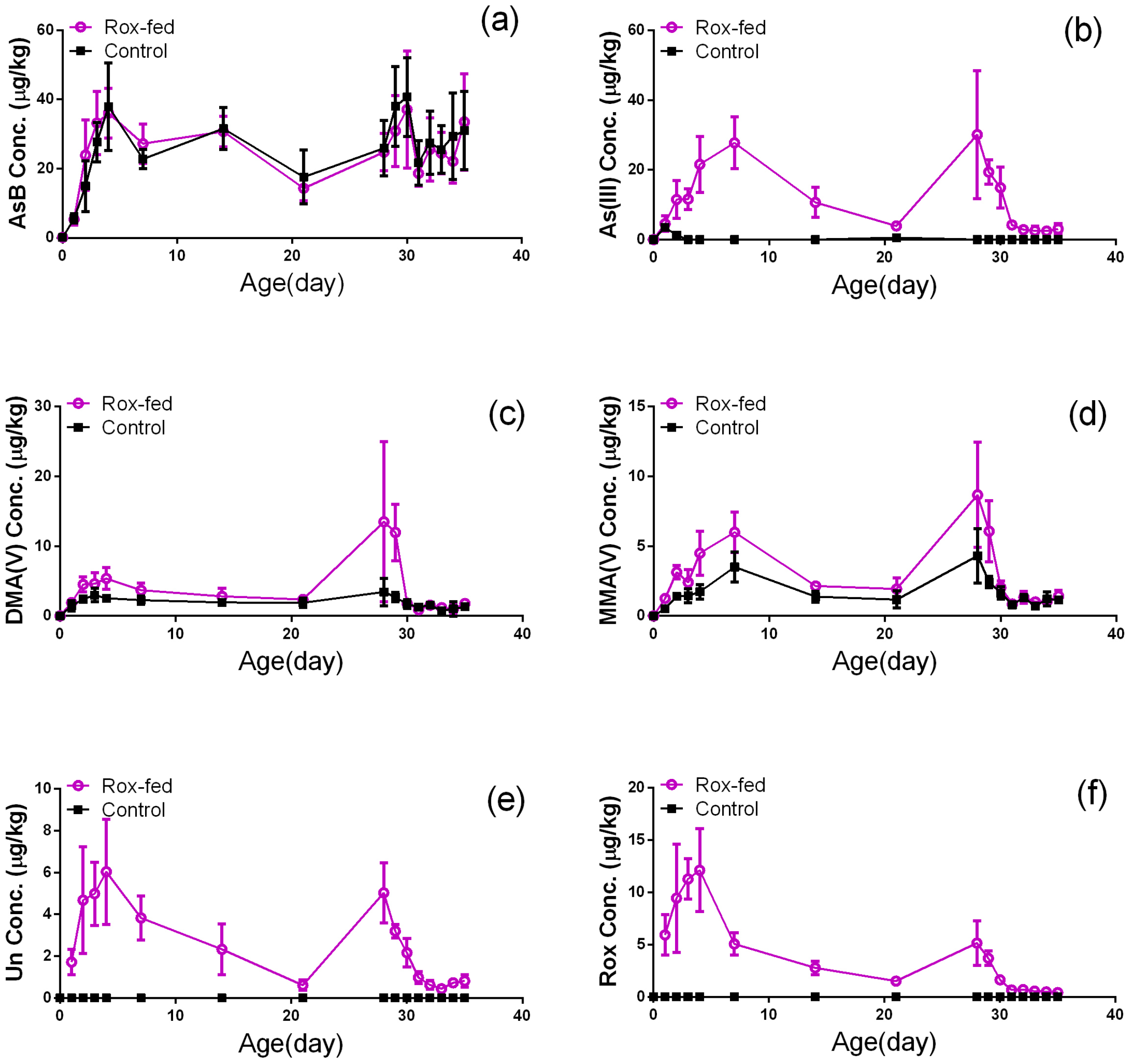

Figure S1. Concentrations of As^{III} (a), DMA^{V} (b), MMA^{V} (c), Unknown arsenic species (d), and Rox (e), without normalization against AsB, in the breast samples of control and Rox-fed chickens over the 35-day feeding period. Data represent mean values and error bars represent one standard deviation from replicate analyses of each of 5-8 chicken samples.

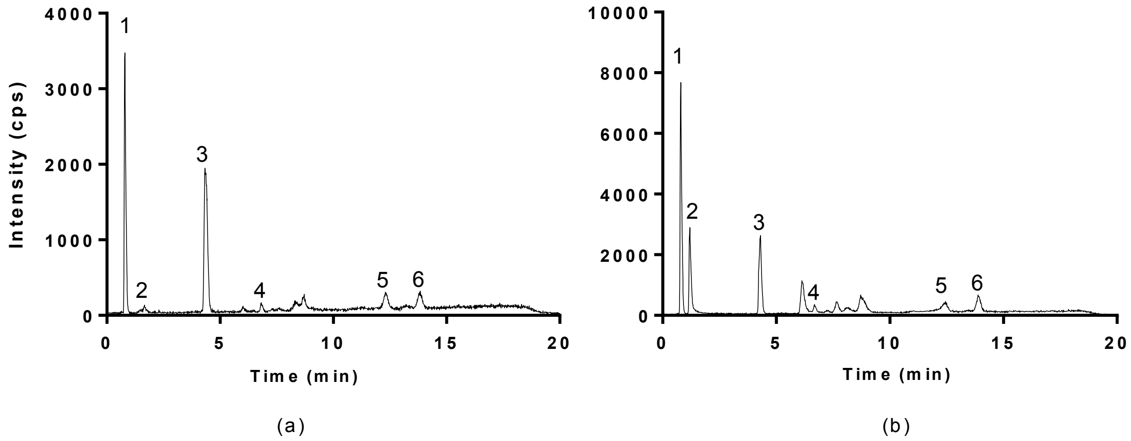

Figure S2. Chromatograms obtained from HPLC-ICPMS analyses of a chicken meat sample after different extraction methods. The peaks labeled with numbers 1 through to 6 correspond to AsB, As^{III}, DMA^{V}, MMA^{V}, Unknown and Rox, respectively. The extraction methods were with water-methanol (left panel) and with papain (right panel). (Liu et al. 2015).

References

Liu Q, Peng H, Lu X, Le XC. 2015. Enzyme-assisted extraction and liquid chromatography mass spectrometry for the determination of arsenic species in chicken meat. Anal Chim Acta 888: 1-9. doi:10.1016/j.aca.2015.05.001.

Peng H, Hu B, Liu Q, Yang Z, Lu X, Huang R, Li X-F, Zuidhof MJ, Le XC. 2014. Liquid chromatography combined with atomic and molecular mass spectrometry for speciation of arsenic in chicken liver. J Chromatog A 1370: 40-49.

United States Environmental Protection Agency (US EPA). 2011. 40 CFR Appendix to part 136 - Definition and procedure for the determination of the method detection limit - revision 1.11. Available: http://www.gpo.gov/fdsys/granule/CFR-2011-title40-vol23/CFR-2011-title40-vol23-part136-appB/content-detail.html [accessed 06 October 2015].
